# Supplementary material for: Transcriptomic Profiling of Quinoa Reveals Distinct Defense Responses to Exogenous Methyl Jasmonate and Salicylic Acid
Source: Plants (Basel). 2025 Jun 3;14(11):1708. doi: 10.3390/plants14111708 (PMC12157332; doi:10.3390/plants14111708)
Supplement: Supplementary file 1 [file plants-14-01708-s001.zip › Supplementary Figures.pdf]

Supplementary information:

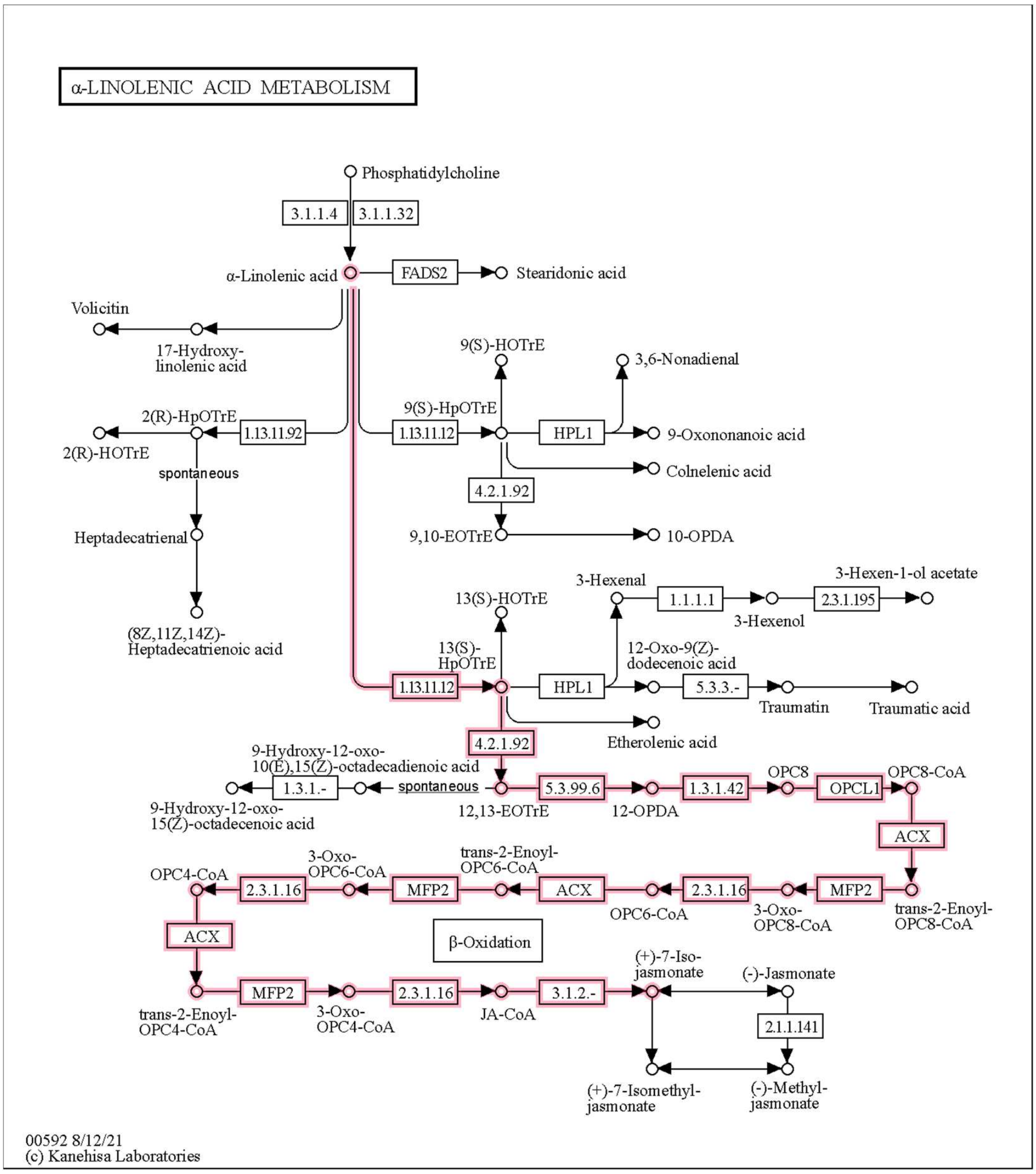

Supplementary Figure S1. Jasmonic acid biosynthesis genes activated by JA. Boxes highlighted in pink shows automatic annotation for quinoa genes differentially induced upon JA treatment by KEGG pathway map viewer. The list of genes can be found in Table 2 and a simplified version can be found in Supplementary Figure S1.

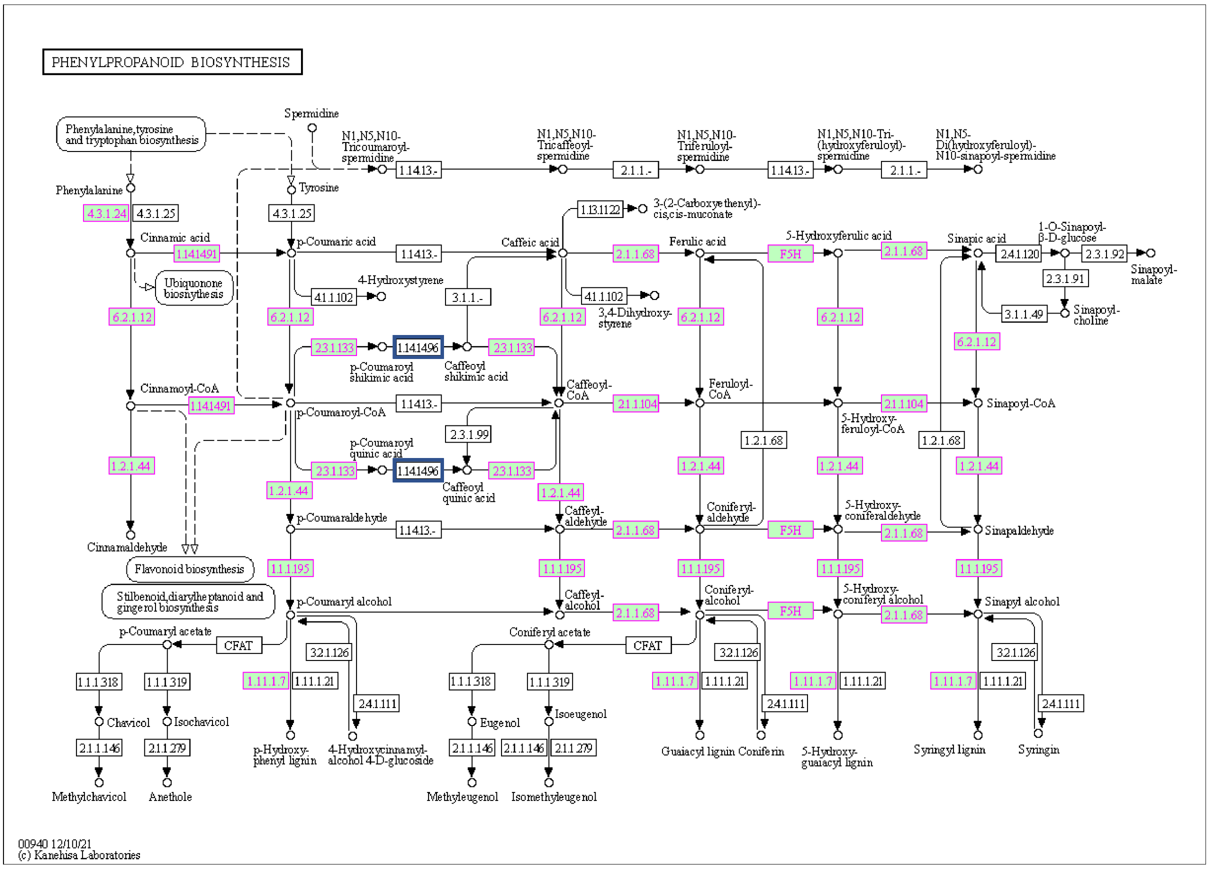

Supplementary Figure S2. Lignin biosynthesis genes activated by JA. Boxes highlighted with pink and green fill show automatic annotation of quinoa genes differentially induced upon JA treatment by KEGG pathway map viewer. Boxes with blue outline show genes (AUR62018397) that showed an at least 1,7-fold increased transcriptomic signal after JA application but not statistically significant. The list of responsive genes can be found in table 3 and a simplified version can be found in Supplementary Figure S2.

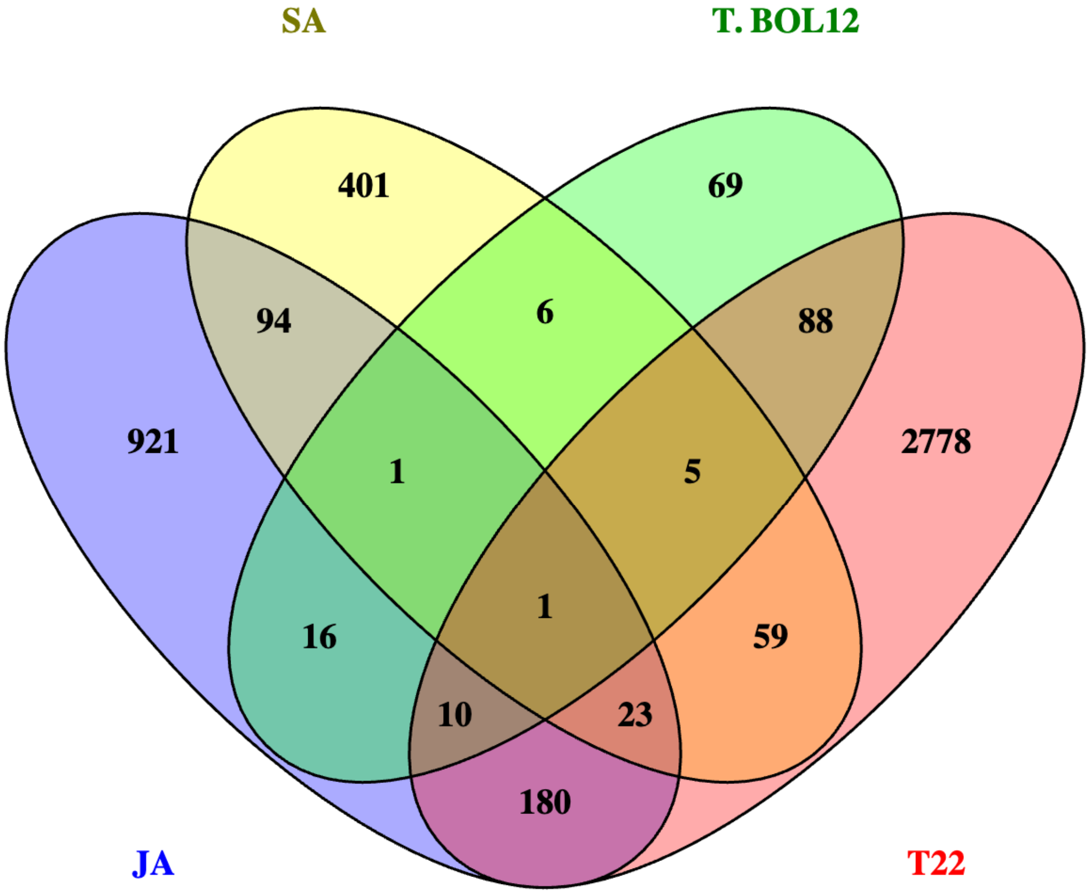

Supplementary Figure S3. Quinoa DE genes upon JA and SA treatment compared to *Trichoderma* spp. treatment.
